# Rho guanine nucleotide exchange factor 39 increases the viability, migration and invasion of clear cell renal cell carcinoma cells via the activation of the AKT/ERK signaling pathway

**DOI:** 10.1590/1678-4685-GMB-2019-0383

**Published:** 2020-11-18

**Authors:** Shuzhong Wang, Yanmei Wang, Chuanyun Wang

**Affiliations:** ^1^ Hubei University of Medicine, Suizhou Hospital, Department of Nephrology, Suizhou, China.; ^2^ Yantai Yuhuangding Hospital, Department of Blood Purification, Shandong, China.; ^3^ Jining No.1 People's Hospital, Department of Urinary Surgery, Shandong, China.

**Keywords:** ARHGEF39, ccRCC, migration, viability, AKT/ERK signaling

## Abstract

We attempted to explore the effect of Rho guanine nucleotide exchange factor 39 (ARHGEF39) on the phenotypes of clear cell renal cell carcinoma (ccRCC) cells and the underlying mechanism. Analyses of the data from The Cancer Genome Atlas (TCGA) illustrated that ARHGEF39 expression was upregulated in ccRCC and high ARHGEF39 expression was correlated with a worse prognosis. The mRNA and protein expression of ARHGEF39 in ccRCC and nontumorigenic cells was measured by qRT-PCR and western blotting, respectively. The results showed that ARHGEF39 expression was upregulated in ccRCC cells compared with nontumorigenic cells. CCK8 and clonogenic assays were used to measure the viability of ccRCC cells after knockdown or overexpression of ARHGEF39. Transwell assays were used to examine the changes in cell motility after alterations in ARHGEF39 expression and treatment with LY294002 (an AKT inhibitor) or PD98059 (an ERK inhibitor). ARHGEF39-mediated changes in the phosphorylation of AKT and ERK were measured by western blotting. The results indicated that ARHGEF39 promoted the viability, migration and invasion of ccRCC cells by regulating the activation of the AKT/ERK signaling pathway. Overall, our research suggested that ARHGEF39 was upregulated in ccRCC and possibly facilitated the malignant development of ccRCC by modulating the AKT/ERK signaling pathway.

## Introduction

Kidney cancer increases progressively with time and seriously affects human health (Owens, 2016). Clear cell renal cell carcinoma (ccRCC) accounts for approximately 60-85% of the pathological type of renal cancer and has the worst prognosis of common epithelial tumors of the kidney (Ljungberg *et al.*, 2015). At present, there is no specific drug for the treatment of ccRCC, and patients with ccRCC often have a poor prognosis. Approximately 30% of patients develop recurrence and metastasis after tumor resection, and few effective treatments are available. Therefore, targeted therapy has become an important strategy for the current ccRCC treatment (Duensing and Hohenfellner, 2016). However, in clinical treatments with targeted therapeutic drugs are only effective in 20% to 40% of patients with ccRCC and the overall survival is still unsatisfactory. Thus, the exploration of genes that are closely related to ccRCC is expected to provide new strategies and theoretical support for tumor gene-targeted therapy.

Rho guanine nucleotide exchange factor 39 (ARHGEF39) is a new member of the Dbl-family of guanine nucleotide exchange factors (GEFs), which are pivotal catalysts of GTPases and are markedly associated with tumor metastasis (Rossman *et al.*, 2005; Goicoechea *et al.*, 2014; Miller *et al.*, 2014; Hennig *et al.*, 2015). ARHGEF39 has been found to be associated with human hepatocellular carcinoma, gastric cancer and nonsmall cell lung cancer (Wang *et al.*, 2012; Cook *et al.*, 2014; Wang *et al.*, 2018; Zhou *et al.*, 2018). According to the research results, ectopic expression of ARHGEF39 is conducive to the proliferation of hepatocellular carcinoma cells by affecting the G2/M phase (Wang *et al.*, 2012; Cook *et al.*, 2014). In gastric cancer, ARHGEF39 has been revealed to promote cancer cell growth and migration through the Akt signaling pathway (Wang *et al.*, 2018). ARHGEF39 promoted non-small cell lung cancer proliferation by stimulating the Rac1-P38-ATF2 signaling pathway and increasing the expression of Cyclin A2, Cyclin D1, and MMP2 (Zhou *et al.*, 2018). These data prompted us to explore whether ARHGEF39 is involved in ccRCC progression and what role it plays.

In our study, we used the data from The Cancer Genome Atlas (TCGA) to explore the expression of ARHGEF39 and the correlation between ARHGEF39 expression and the prognosis of ccRCC. Loss- and gain-of-function assays were conducted to analyze the effect of ARHGEF39 expression on the viability, migration and invasion of ccRCC cells, as well as the activation of the AKT/ERK signaling pathway. To our knowledge, this study describes the expression of ARHGEF39 gene and its biological function in ccRCC cells for the first time, which provides a theoretical basis for further research.

## Material and Methods

### Bioinformatics analysis

The RNA sequencing profiles of 611 samples (including 539 ccRCC tumor samples and 72 normal samples) were obtained from The Cancer Genome Atlas (TCGA) (https://genome-cancer.ucsc.edu/). Perl language software package was used to process and merge all the RNA-Seq data of 611 samples to extract the matrix files. Then the corresponding file of ensembl ID and gene name (Homo_sapiens.GRCh38.89.chr.gtf) was downloaded from the Ensembl website (http://www.ensembl.org/index.html). Using Perl language package, we converted the ensembl IDs into the gene names and obtained the expression profile of the genes in all samples. The differential expression of ARHGEF39 in ccRCC tumor samples and normal samples was analyzed based on these data.

Among the above 539 ccRCC tumor samples, 530 samples possess clinical data, and these data were downloaded for further analysis. These 530 samples were divided into high expression and low expression groups according to the median expression of ARHGEF39. The relationship between ARHGEF39 expression and prognosis was examined by the Kaplan-Meier method, and the differences between two groups were compared by Log-rank test. Chi-square test was used to assess the correlation between ARHGEF39 expression and clinical features. In addition, cox proportional hazards regression analysis was used to investigate whether ARHGEF39 can be used as a predictor of ccRCC prognosis independently.

### Cell culture

The ccRCC cell lines (Caki-1, 786-O, UT33A), as well as nontumorigenic control HK-2 cell line were obtained from the China Center for Type Culture Collection. All the cells were cultured in a 5% CO_2_ incubator at 37 °C. And the culture medium was RPMI-1640 mixed with 10% FBS, 100 IU/mL penicillin and 0.1mg/mL streptomycin. When entering the logarithmic growth phase, the cells were digested and blown into single cell suspension. Then, the cell suspension was seeded in six-well plates for subsequent experiments.

### Cell transfection and treatment

RHGEF39 depleted or overexpressed ccRCC lines were constructed according to the method described previously (Xu *et al.*, 2018) with some modifications. Briefly, the following two different ARHGEF39-siRNA sequences (si-ARHGEF39#1: 5’-TCCATACTATTGGTCAGAAAC-3’ and si-ARHGEF39#2: 5’- CCAATTTGCTGCCAACTCAGA-3’) were designed to knock down ARHGEF39 in ccRCC cells. The control siRNA sequence (si-con) was 5’- CGAACUCACUGGUCUGACC-3’. For overexpression of ARHGEF39, the plasmid pcDNA3.1-ARHGEF39 was transfected into ccRCC cells with pcDNA3.1 empty vector as a control. Cell transfection was performed in the light of the instruction of Lipofectamine 2000 Transfection Kit (invitrogen). When cell confluence approximately achieved 80%, Lipofectamine 2000 (10 μL) and 5 μg siRNA or plasmids were diluted in 500 μL Opti-MEM medium. Then, the mixed solution was added in the 6-well plate and the transfection efficiency was detected after incubating for 48 h.

UT33A cells were treated with the AKT inhibitor LY294002 (20 μM; Cell Signaling Technology, USA) or the ERK inhibitor PD98059 (20 μM; MedChem Express, USA) for 12 h to explore whether inhibition of AKT or ERK would reverse the phenotypes of ccRCC cells induced by ARHGEF39.

### RNA isolation and qRT-PCR assays

After washing all cells twice with PBS, total RNA was extracted with TRIzol reagent in line with manufacturer's instructions. Then the extracted RNA was reverse transcribed to prepare cDNA according to the instructions of PrimeScript RT Reagent Kit. Subsequently, the Real-Time PCR System was utilized to determine the mRNA expression levels of ARHGEF39 in cells via employing the SYBR Premix Ex Taq II kit (TaKaRa). The conditions of qPCR were: 95 °C for 5 min, 40 cycles of (95 °C for 5 s, 60 °C for 34 s), and 72 °C for 30 min. GAPDH was the internal reference to calculate the relative mRNA expression of ARHGEF39 by a 2^−^ΔΔCt method. Triple experiments were performed independently. The primers used in this study were listed as follows: ARHGEF39: F: 5’- GGTTTGTACGGCTTCAGGAAGG-3’, R: 5’-GGACCTGTGTTTTCAGCCAAAGC-3’; GAPDH: F: 5’- TGTGTCCGTCGTGGATCTGA-3’, R: 5’- CCTGCTTCACCACCTTCTTGA-3’.

### Western blotting assays

After 48 hours of transfection, the cells in six-well plates were collected and placed on ice. To extract the proteins, RIPA lysate with protease inhibitor was used. BCA method was used to determine the protein concentration. Then we added about 20 μg protein to each well of a vertical electrophoresis tank after being heated at 95 °C for 5 min. Following that, the protein samples were separated by SDS-PAGE and transferred onto a PVDF membrane. The membrane was blocked in skim milk for 1 h and then incubated with primary antibodies at 4 °C overnight. The primary antibodies used in the current study were list as follows: ARHGEF39 (1:1000, cat.no. ab67211, Abcam), AKT (1:500, cat. no. ab64148, Abcam), p-AKT (1:500, cat.no. ab8932, Abcam), ERK (1:1000, cat.no. ab32537, Abcam), p-ERK (1:1000, cat.no. ab131438, Abcam). Following that, the membrane was rinsed with TBST 35 min and incubated with suitable secondary antibodies at ambient temperature. Then the protein bands were washed and developed with enhanced chemiluminescence western blot detection kit. The gray value was scanned by the QUANTITY ONE software and the relative expression of each protein was calculated with GAPDH as the internal reference.

### CCK-8 assay

Cell counting kit-8 (CCK-8; YEASEN, Shanghai, China) was used to measure the viability of ccRCC cells. After being transfected for 24 h, the cells were detached by trypsin and then cell suspension was prepared. Then, cells were seeded at a density of 110^3^ cells/well with mixed medium (RPMI-1640 + 10% FBS) in a 96-well plate and cultured under standard conditions. Cell viability were detected every 24 h. A total of 10 μL CCK-8 reagent was injected into each well and incubated for 90 min at 37 °C before the detection of optical density (OD) values at 450 nm.

### Transwell invasion and migration assays

The migratory and invasive capacities of ccRCC cells were measured by using transwell chambers. In invasion assay, the chamber was placed in a 24 well plate and incubated with 100 μL of Matrigel gel (Serum-free medium diluted 1:6) for 4-6 hours at 37 °C. After being transfected for 48 h, cell suspension was prepared with serum-free culture. Then 100 μL suspension (110^5^ cells/mL) was plated onto the upper chamber in which Matrigel gel was placed. Next, 500 μL medium containing 10% FBS was placed in the bottom layer. After incubating in a CO_2_ incubator at 37 °C overnight, the cells in upper layer were carefully wiped off. The membranes were then fixed in 4% paraformaldehyde and stained by 0.1% crystal violet after rinsing with PBS. Finally, the invaded cells through the membranes were photographed and counted by using a phase-contrast microscope.

The migration experiment was similar to the above procedure, but the transwell chambers don't need to be coated with Matrigel gel.

### Clonogenic assay

Exponentially growing cells were detached by trypsin to make cell suspension, and 410^2^ cells were then plated in a 60 mm petri dish. Then the cells were cultured at 37 °C for 1-2 weeks and medium was replaced every 3 days. Subsequently, cells were constantly monitored and cell culture was terminated when visible colonies appeared in the culture dish. The cell clones were fixed with 4% paraformaldehyde for 0.5 h after being washed twice with PBS, and then stained by 0.1% crystal violet under ambient condition. After that, the petri dishes were slowly washed with running water and air dried. Then, the number of visible colonies was counted directly.

### Data analysis and statistics

All statistical analyses were conducted using the SPSS 22.0 (IBM SPSS, Armonk, NY, USA) and Graphpad Prism 5.0 software (San Diego, CA, USA). All results were expressed as the mean ± Standard Deviation (SD) and all experiments were performed in triplicate. Shapiro-Wilk test was conducted using SPSS 22.0 software to determine whether the data conforms to a Gaussian distribution. It was regarded as conforming to a Gaussian distribution when p > 0.05. Student's *t*-test was performed to analyze the statistical significant differences between two groups, while one-way Analysis of Variance (ANOVA) with Dunnett's multiple comparisons test was performed to compare the differences among three or more groups. P<0.05 was considered to be statistically significant.

## Results

### ARHGEF39 was up-regulated in ccRCC

**Figure 1:**
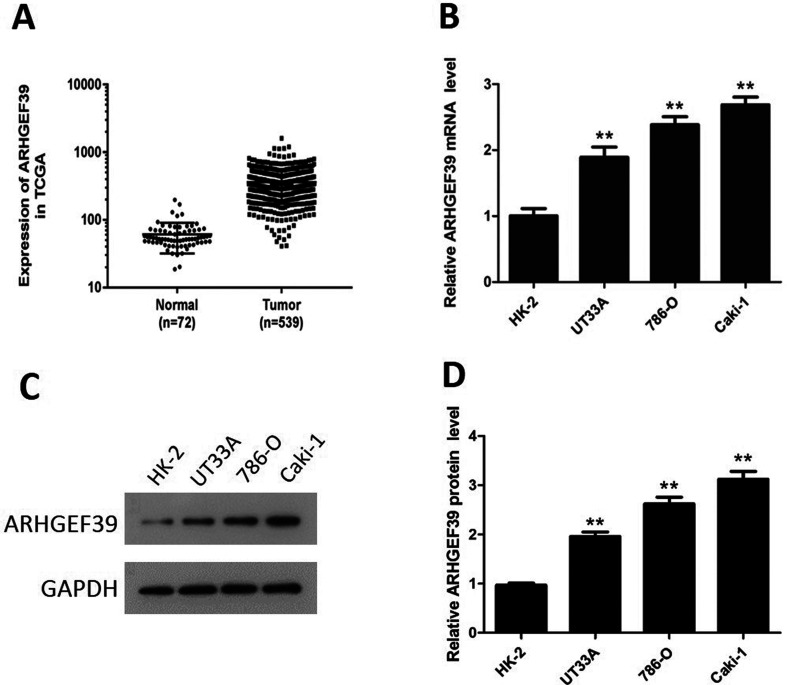
ARHGEF39 expression in ccRCC. A) The expression differences of ARHGEF39 mRNA in ccRCC and normal kidney tissues in TCGA database (tumor n = 539, normal n = 72, p = 0.0005). B) Expression of ARHGEF39 mRNA in UT33A, 786-O, Caki-1 and HK-2 cells (qRT-PCR method). C) Expression of ARHGEF39 protein in UT33A, 786-O, Caki-1 and HK-2 cells (Western blotting method). D) The relative protein level of ARHGEF39 in UT33A, 786-O, Caki-1 and HK-2 cells. **p < 0.01.

The expression of ARHGEF39 in ccRCC and normal tissues was analyzed based on TCGA data. The outcomes illustrated that ARHGEF39 expression was significantly higher in ccRCC tissues than that in normal tissue of kidney (p = 0.0005, Figure 1A). Besides, both mRNA and protein expression levels of ARHGEF39 were significantly higher in ccRCC cell lines (UT33A, 786-O, Caki-1) than nontumorigenic HK-2 cells (p < 0.01, Figure 1B-D).

### Enhanced expression of ARHGEF39 predicted a poor prognosis in ccRCC

The relationship between expression level of ARHGEF39 and clinicopathological characteristics of ccRCC patients was statistically analyzed. The data showed that ARHGEF39 mRNA expression was notably correlated with the pathologic stage (p = 0.003) as well as T stage (p = 0.019), but there was no statistical relevance with Grade of tumor, Pathologic-N, Pathologic-M, gender and age (p > 0.05, Table 1).

**Table 1:** Correlation between ARHGEF39 expression and clinicopathological characteristics of ccRCC patients from TCGA database.

Characteristics	Expression of ARHGEF39		P value
	Low	High	^*^P<0.05
**Age**			0.931
<60	122	123	
[#GTEQ#]60	143	142	
**Gender**			0.856
female	94	92	
male	171	173	
**Grade**			
G1+G2	130	111	0.067
G3+G4	129	152	
**Pathologic-Stage**			0.003*
I+II	178	144	
III+IV	86	119	
**Pathologic-T**			0.019*
T1+T2	183	157	
T3+T4	82	108	
**Pathologic-N**			0.106
N0	124	114	
N1	5	11	
**Pathologic-M**			0.114
M0	224	196	
M1	34	44	

**Figure 2:**
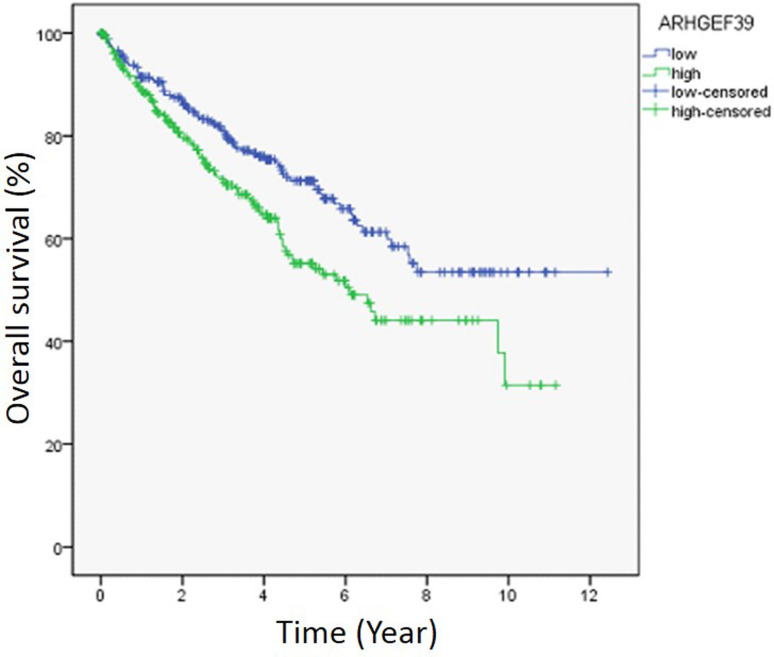
The survival curves of ARHGEF39 expression and prognosis of ccRCC. The relationship between ARHGEF39 expression and prognosis was examined by Kaplan-Meier method, and the differences between two groups were compared by Log-rank test. The ccRCC samples were divided into high expression group (n=265) and low expression group (n=265) according to the median expression of ARHGEF39. Censored data: incomplete data.

Subsequently, the prognostic value of ARHGEF39 in ccRCC was investigated by Kaplan-Meier analysis and log-rank test. It was revealed that the survival rate in high ARHGEF39 mRNA expression group was notably lower than that in the low expression group (p = 0.004, Figure 2).

To further investigate the prognostic value of ARHGEF39 in ccRCC, cox hazards regression analyses were conducted (Table 2). The data of univariate analysis showed that ARHGEF39 expression (HR 1.561, 95% CI: 1.155-2.110, p = 0.004), age (HR 1.781, 95% CI: 1.300-2.439, p = 0.000), tumor grade (HR 2.668, 95% CI: 1.893-3.759, p = 0.000), clinical-stage (HR 3.850, 95% CI: 2.802-5.291, p = 0.000), Pathologic-T (HR 3.152, 95% CI: 2.326-4.272, p = 0.000), Pathologic-M (HR 4.323, 95% CI: 3.163-5.908, p = 0.000) and Pathologic-N (HR 3.411, 95% CI: 1.810-6.428, p = 0.000) were considered to be the prognostic factors that correlated with the overall survival of ccRCC patients. Further multivariate cox analysis demonstrated that tumor grade (HR 1.670, 95% CI: 1.016-2.744, p = 0.043) and Pathologic-M (HR 2.669, 95% CI: 1.572-4.532, p = 0.000) were potential independent indicators for the survival outcomes.

**Table 2:** COX proportional hazards regression analyse for the survival analysis of ccRCC patients from TCGA database.

Variables	Univariate analysis			Multivariate analysis		
	P-value	HR	95% CI	P-value	HR	95%CI
ARHGEF39 expression (high/low)	0.004*	1.561	1.155-2.110	0.168	1.365	0.877-2.124
Clinical-Stage (I+II/III+IV)	0.000*	3.850	2.802-5.291	0.829	1.110	0.428-2.881
Pathologic-T (T1+T2/T3+T4)	0.000*	3.152	2.326-4.272	0.203	1.726	0.745-3.998
Pathologic-M (M0/M1)	0.000*	4.323	3.163-5.908	0.000*	2.669	1.572-4.532
Pathologic-N (N0/N1+N2+N3)	0.000*	3.411	1.810-6.428	0.198	1.597	0.783-3.257
Age (<60/ [#GTEQ#] 60)	0.000*	1.781	1.300-2.439	0.185	1.340	0.869-2.065
Gender (female/male)	0.760	0.953	0.699-1.299			
Grade(G1+G2/G3+G4)	0.000*	2.668	1.893-3.759	0.043*	1.670	1.016-2.744

### Knockdown of ARHGEF39 inhibited ccRCC cell growth, migration, and invasion

**Figure 3:**
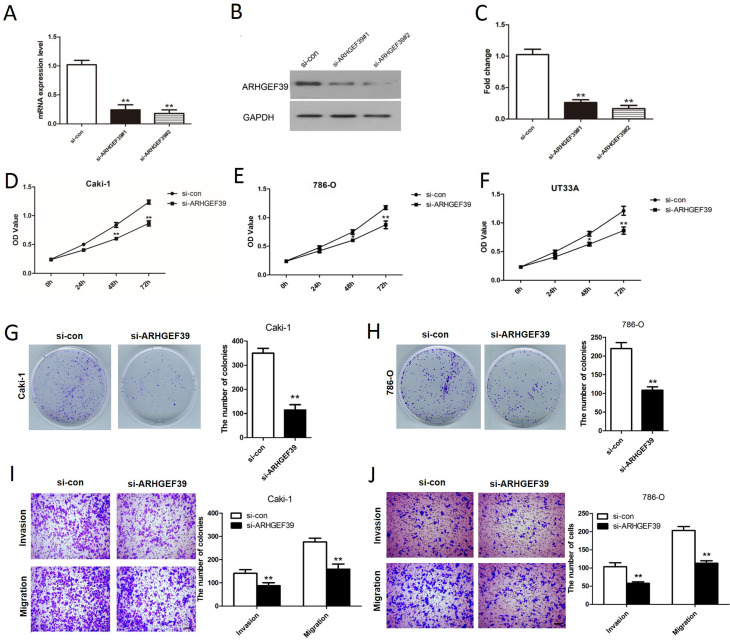
ARHGEF39 knockdown inhibited the viability, migration and invasion of ccRCC cell. A) The mRNA expression of ARHGEF39 in Caki-1 cells transfected with si-con, si-ARHGEF39#1 and si-ARHGEF39#2 were detected by qRT-PCR assay, **p < 0.01. B-C) The ARHGEF39 protein expression in Caki-1 cells transfected with si-con, si- ARHGEF39#1 and si-ARHGEF39#2 were detected by Western blotting assay, **p < 0.01. D-F) The viability of Caki-1, 786-O and UT33A cells with down-regulated ARHGEF39 was measured by CCK8 assay, *p < 0.05, **p < 0.01. G-H) The viability of Caki-1 (G) and 786-O (H) cells in si-ARHGEF39 group was notably lower than that in si-con group which was examined by colony formation assay, **p < 0.01. I-J) The motility of Caki-1 (I) and 786-O (J) cells transfected with si-con or si-ARHGEF39 was detected by Transwell invasion and migration assays, **p < 0.01.

To detect the functional role of ARHGEF39 in ccRCC cells, loss- and gain-of-function assays were performed. Compared with the si-con group, the mRNA and protein expression of ARHGEF39 in ccRCC cells were notably decreased after transfection of si-ARHGEF39 (p < 0.01, Figure 3A-C). The result of CCK8 assay showed that the OD value was significantly decreased after depletion of ARHGEF39 in Caki-1, 786-O and UT33A cells (p < 0.01, Figure 3D-F), indicating that depletion of ARHGEF39 suppressed the viability of ccRCC cells. As the results were consistent in these 3 cell lines and Caki-1 and 786-O cell lines presented the higher expression of ARHGEF39 compared to UT33A cell line, the following loss-of-function assays were performed in Caki-1 and 786-O cells. Clonogenic assay revealed that the number of colonies in si-ARHGEF39 group was notably smaller than that of the si-con group, which further indicated that knockdown of ARHGEF39 notably restricted the viability of Caki-1 and 786-O cells (p < 0.01, Figure 3G-H). In addition, transwell assays were utilized to detect the function of ARHGEF39 on Caki-1 and 786-O cell migration and invasion. The results illustrated that the knockdown of ARHGEF39 notably prevented migration and invasion of the Caki-1 and 786-O cells (p < 0.01, Figure 3I-J).

### Overexpression of ARHGEF39 promoted ccRCC cell growth, migration, and invasion

**Figure 4:**
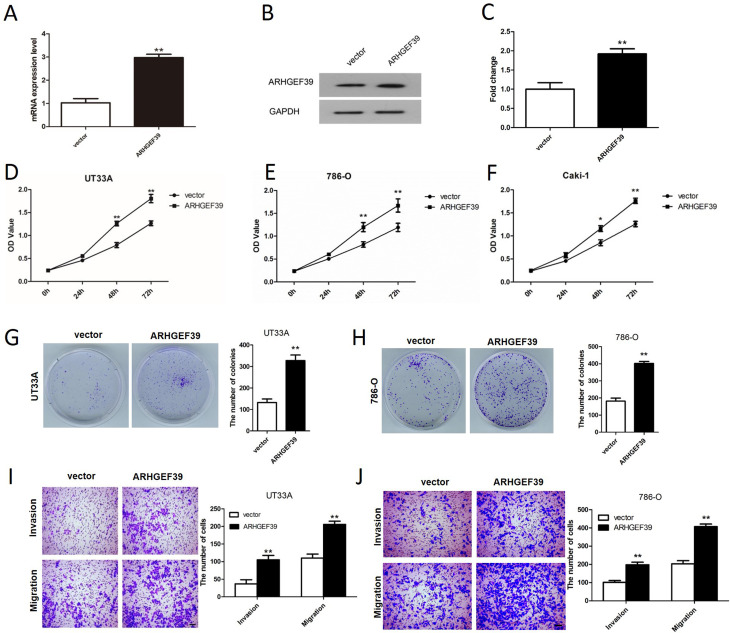
ARHGEF39 overexpression promoted ccRCC cell viability, migration and invasion. A) The mRNA expression of ARHGEF39 in UT33A cells transfected with vector and pcDNA3.1-ARHGEF3 was analyzed by qRT-PCR assay, **p < 0.01. B-C) ARHGEF protein expression in UT33A cells transfected with vector and pcDNA3.1-ARHGEF39 was measured by western blotting assay, **p < 0.01. D-F) The viability of UT33A, 786-O and Caki-1 cells with up-regulated ARHGEF39 was determined by CCK8 assay, **p < 0.01. G-H) Clonogenic assay suggested that the viability of UT33A (G) and 786-O (H) cells in ARHGEF overexpression group was notably increased than that in empty vector group, **p < 0.01. I-J) Transwell invasion and migration assays were carried out to detect the motility of UT33A (I) and 786-O (J) cells transfected with empty vector and pcDNA3.1-ARHGEF3. **p < 0.01.

On the other side, ARHGEF39 was overexpressed in ccRCC cells using overexpression plasmids, and the efficiency of overexpression was also measured by qRT-PCR and western blotting (p < 0.01, Figure 4A-C). Subsequently, CCK-8 assay revealed that overexpression of ARHGEF39 notably promoted the viability of UT33A, 786O and Caki-1 cells (Figure 4D-F). As the results were similar in these three cell lines and UT33A and 786-O cell lines presented a relatively lower ARHGEF39 expression than Caki-1, the following overexpression assays were performed in UT33A and 786-O cells. The results of clonogenic and transwell assays revealed that ARHGEF39 overexpression notably promoted UT33A and 786-O cells viability, migration and invasion (Figure 4G-J).

### ARHGEF39 might affect phenotypes of ccRCC cells by promoting the activation of the AKT/ERK signaling pathway

**Figure 5:**
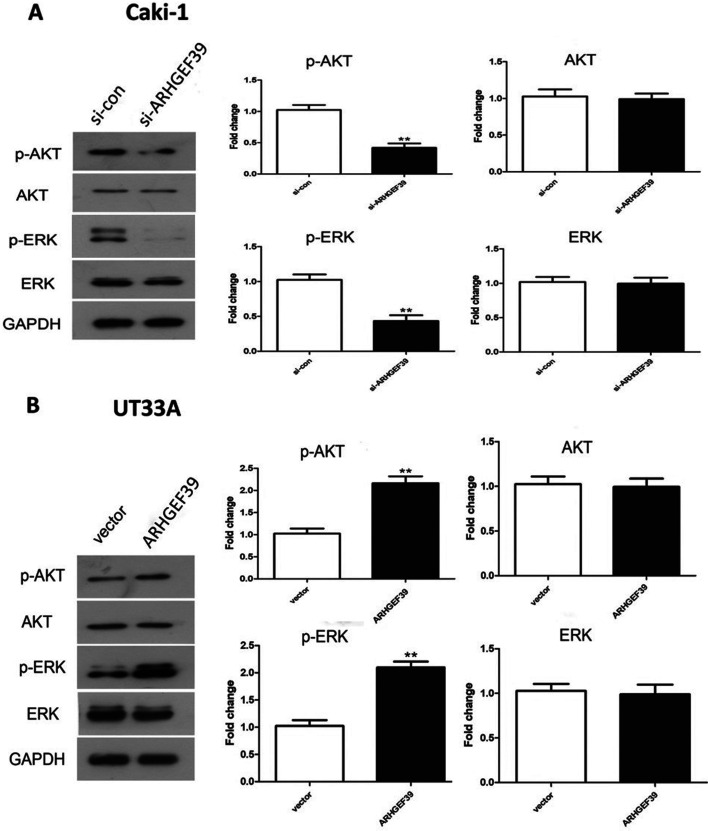
Effect of ARHGEF39 on the expression levels of p-AKT and p-ERK protein in ccRCC cells. A) Effect of ARHGEF39 on the expression levels of p-AKT and p-ERK protein in Caki-1 cells with down-regulated ARHGEF39, **p < 0.01. B) Effect of ARHGEF39 on the expression levels of p-AKT and p-ERK protein in UT33A cells with up-regulated ARHGEF39, **p < 0.01.

**Figure 6:**
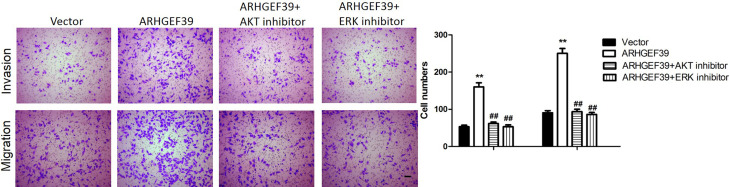
The promoting effect of ARHGEF39 on the migration and invasion was reversed by blocking the activation of AKT and ERK. LY294002, an inhibitor of AKT, was used to block the activation of AKT. PD98059, an inhibitor of ERK, was used to block the activation of ERK. **p<0.01 vs. vector group. ##p<0.01 vs. ARHGEF39 group.

Since the AKT/ERK signaling pathway played important role in tumorigenesis, the effect of ARHGEF39 on the activity of this pathway in ccRCC cells was examined by western blotting. The data showed that the expression of p-AKT and p-ERK proteins were remarkably decreased in the si-ARHGEF39 group, whereas the expression was remarkably increased in the ARHGEF39 overexpression group (p < 0.05, Figure 5. A-B). Moreover, the protein expression of AKT and ERK had no significant change, both in si-ARHGEF39 and ARHGEF39 overexpression groups (p < 0.01, Figure 5 A-B). To explore whether the effect of ARHGEF39 on phenotypes of ccRCC cells was mediated by the AKT/ERK signaling pathway, we treated the ARHGEF39-overexpressed UT33A cells with LY294002 (an AKT inhibitor) or PD98059 (an ERK inhibitor). The results revealed that the promoting effect of ARHGEF39 on the migration and invasion of UT33A cells was reversed by blocking the activation of AKT or ERK (Figure 6). These results suggested that ARHGEF39 might affect the biological phenotypes of ccRCC cells by promoting the activation of the AKT/ERK signaling pathway.

## Discussion

ccRCC is the most common malignancy and its incidence is the highest among kidney cancers (Geissler *et al.*, 2015; Mytsyk *et al.*, 2018). Approximately 20-30% patients with ccRCC have developed metastases at the first diagnosis (Schanza *et al.*, 2017). In recent years, molecular targeted therapy has achieved certain effects in the treatment of ccRCC (Barata and Rini, 2017). Currently, the first-line drugs commonly used are pazopanib and sunotinib, but effectiveness of them is only 20% to 30%, and more than 75% of patients will develop drug resistance within two years (Sternberg *et al.*, 2010; Motzer *et al.*, 2013; Escudier *et al.*, 2014). Therefore, it is significant to screen new biomarkers for ccRCC early diagnosis, prognostic prediction and targeted therapy.

Through previous work, we learned that ARHGEF39 is a new member of the DBL protein family of GEFs, which are notably associated with tumor migration (Liao *et al.*, 2012). GEFs were the major regulators of the Rho family proteins malignant transformation by converting Rho proteins from inactive GDP forms to GTP-like forms (Zhang *et al.*, 2005). ARHGEF39 has been indicated to play a potential carcinogenic role in the development of human hepatocellular carcinoma, gastric cancer and non-small cell lung cancer (Wang *et al.*, 2012, 2018; Zhou *et al.*, 2018). However, ARHGEF39 was found to be absent in extracellular vesicles in urine samples from prostate cancer patients, while it was detected in noncancer samples (Perez *et al.*, 2014). Up to now, there is no report on the expression and prognostic value of ARHGEF39 in ccRCC. Based on these published data, we speculated that this gene may also function in ccRCC.

In the current study, we selected 539 patients with ccRCC and 72 normal controls in the TCGA database to analyze the expression of ARHGEF39. The relationship between ARHGEF39 and pathological grade as well as clinical stage were also evaluated, and the results suggested that ARHGEF39 expression was closely related to the development of ccRCC. In addition, cytological experiments showed that ARHGEF39 overexpression could significantly promote the viability, invasion and migration of ccRCC cells. These data suggests that ARHGEF39 exerts important roles in the viability and motility of ccRCC cells.

The protein kinase AKT and ERK pathway is an important mediator of cell proliferation and tumor development process (Roberts and Der, 2007; Cao *et al.*, 2019). It has been reported that the Rho-family GEFs involved in the calcium, ERK and NF-κB pathways (Costello *et al.*, 1999). In addition, Facio-Genital Dysplasia-5 (FGD5) is an important paralog of ARHGEF39 and it has been revealed that the FGD5 plays a role in VEGF-mediated angiogenesis and regulates the VEGF/PI3 kinase/Akt pathway (Nakhaei-Nejad *et al.*, 2012; Farhan *et al.*, 2017). However, the effect of ARHGEF39 on the AKT/ERK signaling pathway is still not clear. Therefore, this study investigated whether ARHGEF39 regulates the function of ccRCC by affecting the AKT/ERK signaling pathway. The outcomes revealed that high expression of ARHGEF39 promoted the phosphorylation of AKT and ERK. Furthermore, the promoting effect of ARHGEF39 on the migration and invasion of UT33A cells was reversed by blocking the activation of AKT or ERK. The above results indicated that ARHGEF39 possibly affected the phenotypes of ccRCC cells by stimulating the activation of the AKT/ERK signaling pathway. To a certain extent, ARHGEF39 is expected to be a new gene target for early diagnosis and treatment of lacks. However, the current research is not deep enough and lacks experiments in *vivo.* In our subsequent experiments, the molecular mechanism of the ARHGEF39 regulation on ccRCC cells and the *in vivo* experiments will be further studied.

In conclusion, our study suggested that ARHGEF39 exerts a promoting role in the viability, migration and invasion of ccRCC cells, which is probably realized via regulating the AKT/ERK signaling pathway. More in-depth researches including *in vivo* assays on the function and mechanism of ARHGEF39 in ccRCC are needed in the future to further verify the exact role of ARHGEF39.
